# An assessment of cardiovascular disease hospitalizations and disparities by race in patients with rheumatic disease hospitalizations in Alaska, 2015–2018

**DOI:** 10.1186/s41927-024-00377-8

**Published:** 2024-02-18

**Authors:** Elizabeth D. Ferucci, Peter Holck

**Affiliations:** Research Services Department, Division of Community Health Services, Alaska Native Tribal Health Consortium, 3900 Ambassador Drive, 2nd floor Anchorage, 99508 Anchorage, AK USA

**Keywords:** Cardiovascular Disease, Rheumatic diseases, Hospitalization, Health Care disparities

## Abstract

**Background:**

There is an increased risk of cardiovascular disease in people with many rheumatic diseases. The primary objective of this study was to evaluate cardiovascular disease hospitalizations in Alaska for people with and without a rheumatic disease diagnosis and assess disparities by race, with a focus on Alaska Native and American Indian people.

**Methods:**

This study used the Alaska Health Facilities Data Reporting Program data on inpatient hospitalizations from 2015 to 2018. We identified people with a rheumatic disease diagnosis based on any hospitalization with a set of rheumatic disease diagnoses and compared them to people hospitalized but without a rheumatic disease diagnosis. We determined the odds of cardiovascular disease hospitalization by rheumatic disease diagnosis and assessed the influence of race and other factors, using univariate analyses and multivariable models.

**Results:**

People with a rheumatic disease diagnosis other than osteoarthritis had higher odds of cardiovascular disease hospitalization. The odds ratio was highest in people with gout compared to other rheumatic diseases. In multivariable models, there was an interaction between race and rheumatic disease status. Specifically, having gout increased the odds of cardiovascular disease hospitalization for people of all races, while having a rheumatic disease other than gout or osteoarthritis increased the odds of cardiovascular disease hospitalization in Alaska Native/American Indian people but not in people of other races.

**Conclusions:**

The association between rheumatic disease status and cardiovascular disease hospitalization in Alaska varied by type of rheumatic disease and race. This adds substantially to the literature on associations between rheumatic disease and cardiovascular disease in Indigenous North American populations.

**Supplementary Information:**

The online version contains supplementary material available at 10.1186/s41927-024-00377-8.

## Background

There is an increased risk of cardiovascular disease associated with many rheumatic diseases. A systematic review and meta-analysis found that the increased risk of myocardial infarction (MI) was consistently found in population-based studies of rheumatoid arthritis (RA), gout, psoriatic arthritis, and osteoarthritis [1]. In addition, there is an increased risk of heart failure in RA [2]. Systemic lupus erythematosus (SLE), while less common than RA, is well-established to increase the risk of cardiovascular disease compared to the general population, with an especially high relative risk of cardiovascular disease in young women [3, 4]. The increased risk of myocardial infarction, heart failure and other cardiovascular disease is partially explained by traditional risk factors but is also influenced by systemic inflammation in rheumatic disease, glucocorticoid use, and other disease or medication-related factors [1, 3, 5]. 

Several studies have evaluated cardiovascular disease hospitalizations associated with rheumatic diseases, as well as trends over time. From 2000 to 2014, a study in the U.S. found that hospitalizations for people with RA had a decrease in proportion due to acute MI, increase in proportion due to heart failure, and stable proportion due to stroke, and that the absolute number of hospitalizations increased [6]. In patients with SLE in the U.S., the rates of acute MI and stroke hospitalization increased from 1996 to 2012, while rates decreased in the general population over that period [7]. 

Few studies have investigated health disparities in cardiovascular disease in people with rheumatic disease. A recent systematic review found a paucity of data on atherosclerotic cardiovascular disease in RA stratified by race or ethnicity [8]. High rates of some rheumatic diseases such as RA, SLE, and spondyloarthritis have been reported in Indigenous North American (INA) populations [9]. The Alaska Native population has historically had low rates of both diabetes and cardiovascular disease [10]. While diabetes prevalence remains low in Alaska Native people, rates of prediabetes are comparable to the general population and diabetes prevalence is increasing [11, 12]. Mortality due to coronary heart disease remained low in a recent study in Western Alaska, but stroke rates and mortality were high [13]. 

Our overall study was designed to evaluate the impact of rheumatic disease on the Alaska Native/American Indian (AN/AI) population in Alaska. We previously described overall hospitalizations and hospitalized infections associated with rheumatic disease in the population of Alaska [14, 15]. In the analysis presented herein, we focused on hospitalizations for cardiovascular disease in people with or without a rheumatic disease diagnosis during a hospitalization using the same dataset and similar methods to our previous publications. We included all hospitalizations in Alaska from 2015 to 2018 and examined odds of cardiovascular disease hospitalization by rheumatic disease status, AN/AI race, and other factors. We hypothesized that people with rheumatic disease diagnoses would be more likely to have any hospitalization for cardiovascular disease and that this difference would be more pronounced for AN/AI people, who were expected to have lower likelihood of cardiovascular disease hospitalizations at baseline.

## Methods

### Study population and data source

We obtained data for this study from the Alaska Inpatient Database of the State of Alaska Health Facilities Data Reporting Program (HFDR). The HFDR collects mandated reports on discharge data from hospitals and health care facilities in Alaska. While the HFDR contains some outpatient data, only inpatient data reporting is mandated and we used only the inpatient data for this study because of the limited availability of outpatient data. The dataset is de-identified for use in research, population health assessment or health care operations. Data may not be linked with other datasets to identify cohorts of patients. Within the dataset, patients have unique identifiers which allow tracking of the same person across multiple hospital admissions during the study period. The dataset includes demographics (age, gender, race, region of residence) and hospital discharge diagnoses (primary and all listed, with up to 29 secondary diagnoses allowed) as well as characteristics of hospital encounters (length of stay, discharge status, and in-hospital mortality). For our data analysis, we specified an individual person’s race by the category listed in the HFDR, which is determined by the submitting health care facility and typically collected from the patient by self-report upon registration at the facility. Options for race in the HFDR dataset included AN/AI, White, Black, Asian, Native Hawaiian/Pacific Islander, or Other. The HFDR does not include a multiracial category. For comparisons of race, we excluded persons with missing race in the HFDR. We were interested in differences between AN/AI and non-AN/AI people, and because of observed differences between White and other races, we chose to specify three categories for race: AN/AI, White, and Other (which combined Black, Asian, Native Hawaiian/Pacific Islander, and Other as listed in HFDR). As the HFDR dataset from the years of interest only allows reporting male or female gender, we were unable to include other gender categories. Information on individual-level social determinants of health including income, medical insurance status, and zip code were either not available in the dataset for this study or not allowable based on our data use agreement.

### Case definitions

Eligibility criteria included Alaska residence, having any hospitalization in Alaska from 2015 to 2018, and being age 18 years and older as of the hospital encounter. We excluded non-residents of Alaska because our primary focus was on Alaska residents and we expected the non-residents’ characteristics to differ. Within the eligible population, we identified people with any rheumatic disease diagnosis (primary or alternate) during a hospitalization from 2015 to 2018 and a comparison group of all people with a hospitalization during that time period but no rheumatic disease diagnosis, as described previously [14, 15]. We identified diagnoses of rheumatic disease using International Classification of Diseases (ICD)-9 and ICD-10 codes as described previously [14, 15]. These included nine categories of conditions (osteoarthritis, gout, RA, spondyloarthritis, SLE or mixed connective tissue disease (MCTD), systemic sclerosis, vasculitis, Sjögren syndrome, and inflammatory myopathy). While we were unable to obtain any identifiable data for the purposes of case validation, these ICD-9 and ICD-10 codes were selected based on typical coding for these conditions and have been consistent across all components of this study. In our analysis, we considered three categories of rheumatic disease diagnosis based on expected differences in the likelihood of cardiovascular disease hospitalizations: (1) osteoarthritis alone; (2) gout with or without another rheumatic disease; and (3) all other rheumatic diseases.

We identified cardiovascular disease hospitalizations based on having a primary diagnosis of one of a set of cardiovascular disease diagnoses. These were defined by sets of ICD-9 or ICD-10 codes, as listed in Supplementary Table 1. We grouped cardiovascular diseases into five categories: acute myocardial infarction (MI); cerebrovascular disease (stroke); heart failure and cardiomyopathy (CHF); coronary atherosclerosis and other heart disease (ASC); and hypertension complications (HTN).

### Statistical analysis

We determined the proportion of people with a rheumatic disease diagnosis in each of the three categories (osteoarthritis alone, gout, and all other rheumatic diseases) with any cardiovascular disease hospitalization during the study period and compared them to people with no stated rheumatic disease diagnosis. In univariate analysis, we compared the proportion of people with cardiovascular disease hospitalizations by patient characteristics including demographics (age, gender, race, urban vs. rural residence), comorbidities (Deyo-Charlson comorbidity index based on ICD-9 and ICD-10 codes [16]), and total number of hospitalizations during the study period. We also compared characteristics of cardiovascular disease hospitalizations between those with or without rheumatic disease diagnoses, including length of stay, discharge to home vs. other, and in-hospital mortality. Characteristics of hospitalizations were analyzed as dichotomous variables, with length of stay categorized as less than or equal to 3 days, vs. greater than 3 days, in order to be consistent with other analyses of arthritis hospitalization data [17]. 

We examined whether the likelihood of cardiovascular disease hospitalization varied by type of rheumatic disease, including our categories as described as well as for each individual rheumatic disease. We also investigated if variations existed in the proportion with cardiovascular disease hospitalization by the type of cardiovascular disease leading to hospitalization. Because race appears to be associated with both cardiovascular disease hospitalization and rheumatic disease state, we examined differences by race as well (using categories described above: AN/AI, White, and Other).

We constructed multivariable logistic models to adjust for differences in patient characteristics between categories of rheumatic disease, compared to no rheumatic disease, with or without a cardiovascular disease hospitalization. We considered models with and without interaction terms and included interaction terms in our final models for a more complete specification of the effects of covariates.

In analyses by race, we excluded people of unknown race (accounting for less than 5% of hospitalizations). We also excluded people whose only hospitalizations were pregnancy-related. A small number of people contributed data for only for part of the four-year study period, as the period of eligibility for inclusion started at age 18 and ended if in-hospital death occurred. A sensitivity analysis did not indicate meaningful differences in characteristics of interest of the few people with a shorter eligibility period (also less than 5% overall).

## Results

In the cohort of people with no identified rheumatic disease diagnosis there were 7845 CVD hospitalizations (12.7%), compared to 1076 (10.2%) in people with osteoarthritis only, 870 (30.2%) in people with gout, and 341 (14.0%) in people with other rheumatic diseases. A comparison of cardiovascular disease hospitalizations in people with osteoarthritis alone, gout, other rheumatic diseases, or no rheumatic disease diagnosis is shown in Table 1. For patient characteristics including age group, gender, race, type of residence, and Deyo-Charlson comorbiditiy index, the denominators for Table 1 represent the number of people in the cohort for that column who have the characteristic of interest.

**Table 1:** Number (Proportion) of Patients with any Cardiovascular Disease (CVD) Hospitalization by Patient Characteristics of Patients with any Hospital-Based Diagnosis of Osteoarthritis, Gout, or Other Rheumatic Disease, Compared to No Rheumatic Disease Diagnosis

Characteristic	Osteoarthritis Only (of 10,516 people)	Gout (of 2880 people)	Other Rheumatic Disease^a^ (of 2436 people)	No Rheumatic Disease Diagnosis (of 61,896 people)
Any CVD hospitalization	1076 (10.2%)*	870 (30.2%)*	341 (14.0%)	7845 (12.7%)
Age group				
18–39	8 (3.0%)	22 (21.8%)*	16 (6.0%)*	355 (2.2%)
40–64	294 (6.7%)*	277 (28.4%)*	126 (11.2%)	3428 (13.0%)
65 or older	774 (13.3%)*	571 (31.7%)*	199 (19.0%)	4062 (21.4%)
Gender				
Female	574 (9.8%)	252 (32.0%)*	221 (12.5%)*	2970 (10.1%)
Male	502 (10.8%)*	618 (29.5%)*	120 (17.8%)	4875 (15.1%)
Race				
White	686 (8.9%)*	546 (28.6%)*	183 (13.8%)	5052 (13.5%)
AN/AI	247 (14.8%)*	82 (29.9%)*	117 (14.1%)*	1448 (9.3%)
Other	136 (15.3%)	231 (36.6%)*	36 (16.0%)	1157 (16.4%)
Type of residence				
Urban	726 (10.1%)*	609 (29.6%)*	210 (13.8%)	5116 (12.7%)
Rural	350 (10.5%)*	261 (31.7%)*	131 (14.4%)	2724 (12.6%)
Deyo-Charlson Comorbidity Index				
0	42 (0.9%)*	19 (4.8%)*	9 (4.1%)	440 (1.6%)
1–2	259 (7.2%)*	118 (15.4%)	55 (4.8%)*	3085 (15.3%)
>=3	775 (31.3%)	733 (42.7%)*	277 (25.6%)*	4320 (30.9%)

a: Other Rheumatic Disease includes people with a diagnosis of rheumatoid arthritis, lupus/MCTD, spondyloarthritis, vasculitis, Sjögren syndrome, systemic sclerosis, and/or myopathy. Some persons included also have osteoarthritis in addition to one or more of these conditions. Persons with gout are excluded from this category regardless of other conditions

* *p* < 0.01 for comparison with people with no rheumatic disease diagnosis

Overall, the proportion of people with a cardiovascular disease hospitalization was higher in people with gout or another rheumatic disease diagnosis than among those with no rheumatic disease diagnosis, while it was lower among people with osteoarthritis alone. This increase in proportion of cardiovascular disease hospitalizations for people with gout compared to no rheumatic disease diagnosis was present in all age groups. In contrast, only people in the youngest age group (age 18–39 years) had a higher proportion of cardiovascular disease hospitalizations in the setting of other non-osteoarthritis rheumatic disease diagnoses compared to no rheumatic disease diagnosis (6.0% vs. 2.2%, respectively). Men were more likely to have a cardiovascular disease hospitalization than women, regardless of rheumatic disease state. In people without any rheumatic disease diagnosis, AN/AI people had a lower proportion of cardiovascular disease hospitalizations than persons of White or Other race. The increased proportion of cardiovascular disease hospitalizations was found in all races for gout when compared to no rheumatic diseases (range 28.6–36.6%, compared to 9.3–16.4%), but only among AN/AI people for other non-osteoarthritis rheumatic diseases (14.1% vs. 9.3%).

Characteristics of CVD hospitalization encounters are presented in Table 2. For characteristics of CVD hospitalizations, denominators for length of stay, discharge status, and mortality are all CVD hospitalizations for people in the column cohort, with each of these outcomes presented as dichotomous. People with gout or osteoarthritis had a longer length of stay of cardiovascular disease hospitalization but no significant difference in in-hospital mortality compared to those without a rheumatic disease diagnosis. There was no difference in in-hospital mortality based on rheumatic disease state.

**Table 2:** Characteristics of CVD Hospitalizations for Patients with Hospital-Based Diagnosis of Osteoarthritis, Gout, or Other Rheumatic Disease, Compared to No Rheumatic Disease Diagnosis

Characteristic	Osteoarthritis Only	Gout	Other Rheumatic Disease^a^	No Rheumatic Disease Diagnosis
Length of stay of CVD hospitalization				
<=3 days	794 (48.7%)	670 (44.4%)	253 (51.0%)	5601 (53.1%)
>3 days	835 (51.3%)**	838 (55.6%)**	243 (49.0%)	4953 (46.9%)
Discharge status				
To home	1192 (78.6%)	1167 (81.2%)	381 (81.4%)	8099 (82.0%)
Other than home	324 (21.4%)**	271 (18.8%)	87 (18.6%)	1777 (18.0%)
In-hospital mortality				
Discharge home	1192 (94.5%)	1167 (94.8%)	381 (93.8%)	8099 (93.4%)
Death in hospital	70 (5.5%)	64 (5.2%)	25 (6.2%)	574 (6.6%)

a: Other Rheumatic Disease includes people with a diagnosis of rheumatoid arthritis, lupus/MCTD, spondyloarthritis, vasculitis, Sjögren syndrome, systemic sclerosis, and/or myopathy. Some persons included also have osteoarthritis in addition to one or more of these conditions. Persons with gout are excluded from this category regardless of other conditions

** *p* < 0.01 for odds ratio comparing odds comparing two categories of discharge type or length of stay among people the rheumatic disease diagnosis to those with no rheumatic disease diagnosis

Table 3 shows the comparison of the odds of cardiovascular disease hospitalization across different types of rheumatic disease, with all ages, genders, and races combined.

**Table 3:** Odds of Cardiovascular Disease (CVD) Hospitalization by Type of Rheumatic Disease for People of All Races in Alaska, 2015–2018

Type of Rheumatic Disease*	Number with this Rheumatic Disease	Number (%) with Any CVD Hospitalization	Odds Ratio (95% Confidence Interval)	*p*-value
None	61,896	7845 (12.7%)	Reference	
Osteoarthritis	11,943	1359 (11.4%)	0.88 (0.83–0.94)	< 0.01
Gout	2880	870 (30.2%)	2.98 (2.74–3.24)	< 0.01
Rheumatoid arthritis	1710	246 (14.4%)	1.16 (1.01–1.33)	0.04
Lupus or MCTD	384	68 (17.7%)	1.48 (1.12–1.93)	< 0.01
Spondyloarthritis	314	33 (10.5%)	0.81 (0.55–1.16)	0.28
Vasculitis	165	45 (27.3%)	2.58 (1.79–3.67)	< 0.01
Sjögren syndrome	145	15 (10.3%)	0.79 (0.43–1.36)	0.48
Systemic sclerosis	72	15 (20.8%)	1.81 (0.95–3.25)	0.07
Myopathy	45	8 (17.8%)	1.49 (0.60–3.25)	0.41

*One patient can be in multiple rows, if multiple types of rheumatic disease. Therefore, the number of patients with a diagnosis of osteoarthritis is higher in Table 2 than in Table 1, as Table 1 restricts to diagnosis of osteoarthritis alone

**Fig. 1:**
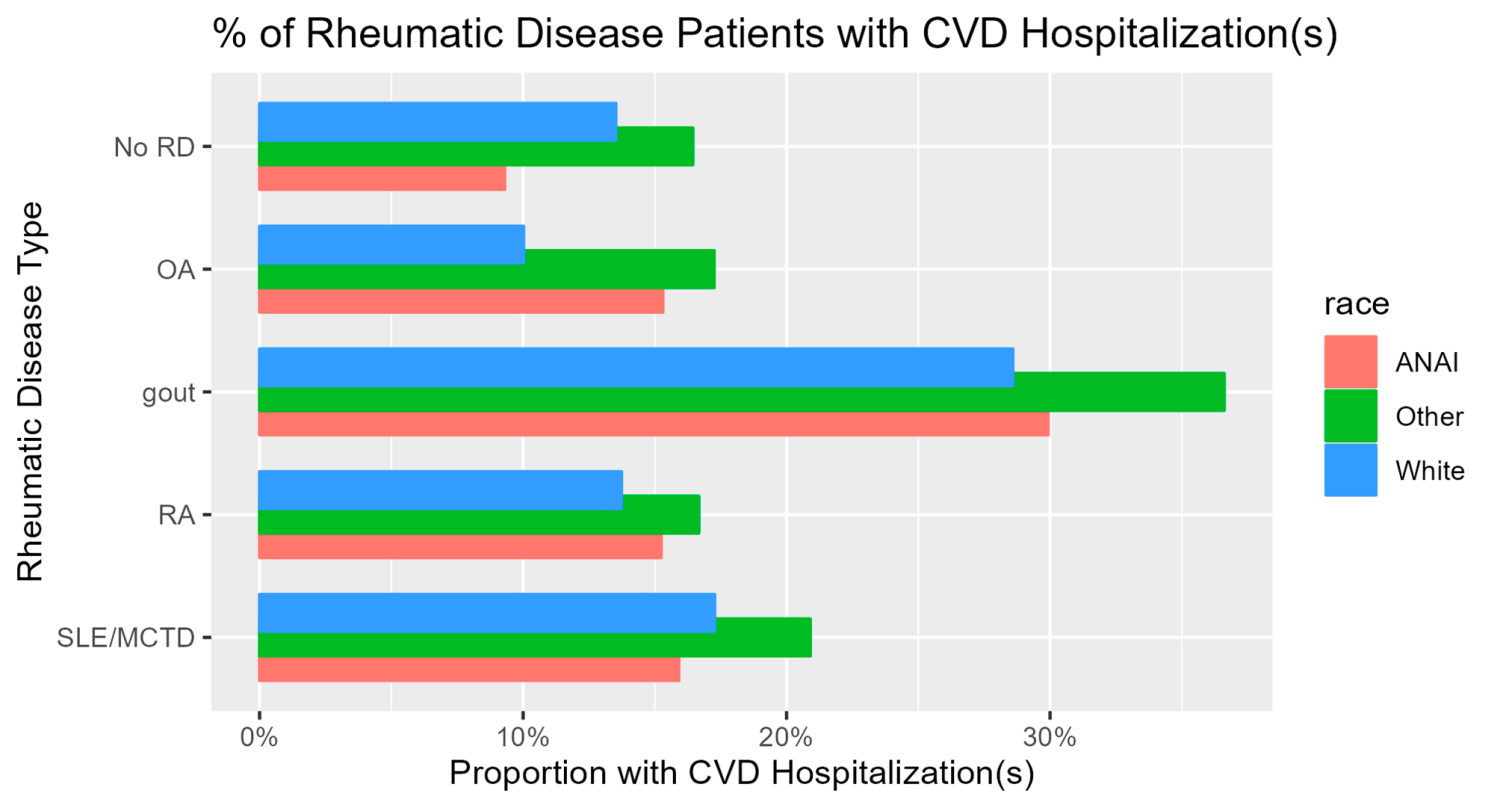
Percent of rheumatic disease patients with any cardiovascular disease hospitalization by rheumatic disease type and by race. This figure excludes spondyloarthritis, vasculitis, Sjögren syndrome, systemic sclerosis and myopathy because there were fewer than 50 people in each of these groups with a cardiovascular disease hospitalization. Comparisons by race were statistically significant (at *p* < 0.01) for all rheumatic disease types displayed except rheumatoid arthritis (*p* = 0.53) and SLE/MCTD (*p* = 0.71). Abbreviations: RD (rheumatic disease), OA (osteoarthritis), RA (rheumatoid arthritis), SLE/MCTD (systemic lupus erythematosus or mixed connective tissue disease), AN/AI (Alaska Native or American Indian)

The conditions with the highest odds of cardiovascular disease hospitalization were gout (odds ratio (OR) 2.98) and vasculitis (OR 2.58). Of the four most common rheumatic diseases (osteoarthritis, gout, RA, and SLE/MCTD), in these univariate analyses, the only condition associated with lower odds of cardiovascular disease hospitalization compared to people with no rheumatic disease diagnosis was osteoarthritis (OR 0.88). Figure 1 shows the proportion of people with any cardiovascular disease hospitalization by type of rheumatic disease (no rheumatic disease and the four most common rheumatic diseases) and by race.

Comparisons by race showed significant differences for all rheumatic disease categories except RA and SLE/MCTD. In individuals without rheumatic disease, AN/AI people had the lowest proportion of cardiovascular disease hospitalizations, while for osteoarthritis and gout, the proportion of hospitalizations in AN/AI people was intermediate between those of White race (lowest) and Other race (highest).

The proportion of cardiovascular disease hospitalization by type of cardiovascular disease and type of rheumatic disease are shown in Table 4 (all ages, genders and races combined).

**Table 4:** Number (Proportion) of Patients with any Cardiovascular Disease (CVD) Hospitalization by Type of CVD of Patients with Osteoarthritis (OA) Only, Gout, Other Rheumatic Disease (RD) Hospitalization, or No RD Hospitalization

Type of CVD	Osteoarthritis Only, n (%) with one or more hospitalizations for this CVD type	Gout, n (%) with one or more hospitalizations for this CVD type	Other RD,^a^ n (%) with one or more hospitalizations for this CVD type	No RD Diagnosis, n (%) with one or more hospitalizations for this CVD
Any CVD hospitalization	1076 (10.2%)*	870 (30.2%)*	341 (14.0%)	7845 (12.7%)
Acute Myocardial Infarction (MI)	296 (2.8%)*	230 (8.0%)*	96 (3.9%)	2615 (4.2%)
Stroke	343 (3.3%)*	197 (6.8%)*	103 (4.2%)	2486 (4.0%)
Heart Failure	353 (3.4%)	406 (14.1%)*	126 (5.2%)*	2119 (3.4%)
Coronary atherosclerosis and other heart disease	180 (1.7%)	111 (3.9%)*	31 (1.3%)	960 (1.6%)
Hypertension complications other than heart disease	44 (0.4%)	72 (2.5%)*	18 (0.7%)	317 (0.5%)

a: Other Rheumatic Disease includes people with a diagnosis of rheumatoid arthritis, lupus/MCTD, spondyloarthritis, vasculitis, Sjögren syndrome, systemic sclerosis, and/or myopathy. Some persons included also have osteoarthritis in addition to one or more of these conditions. Persons with gout are excluded from this category regardless of other conditions

*p-value < 0.01 for comparison to persons with no RD diagnosis

Hospitalization for cardiovascular disease was most common in people with gout, and all types of cardiovascular disease hospitalizations were more common in people with gout compared to people with no rheumatic disease diagnosis. Among people with other rheumatic diseases, only heart failure hospitalizations were more common than occurred in people with no rheumatic disease diagnosis. People with osteoarthritis had a lower likelihood of cardiovascular disease hospitalization overall and lower likelihood specifically for acute MI or stroke hospitalization compared to people without a rheumatic disease.

In Table 5, we present multivariable models of factors associated with cardiovascular disease hospitalizations in people with or without a rheumatic disease diagnosis, by type of rheumatic disease.

**Table 5:** Multivariable Model of Associations with Cardiovascular Disease (CVD) Hospitalization for People with Osteoarthritis (OA), Gout, or Other Rheumatic Disease (RD) Hospitalization Compared to No RD Hospitalization

	Model for OA vs. No RD	Model for Gout vs. No RD	Model for Other RD vs. No RD
Predictor Variable	Odds Ratio (95% Confidence Interval) of CVD Hospitalization	Odds Ratio (95% Confidence Interval) dof CVD Hospitalization	Odds Ratio (95% Confidence Interval) of CVD Hospitalization
Age			
Under 65 years	Reference	Reference	Reference
65 years or older	2.66 (2.53–2.79)	2.56 (2.44–2.69)	2.67 (2.54–2.81)
Total number of hospitalizations for any condition (for every increase by 1)	1.17 (1.16–1.18)	1.16 (1.15–1.18)	1.15 (1.14–1.16)
Death during hospitalization			
No	Reference	Reference	Reference
Yes	1.50 (1.38–1.62)	1.43 (1.31–1.55)	1.43 (1.32–1.56)
Gender			
Female	Reference	Reference	Reference
Male	1.59 (1.51–1.66)	1.60 (1.52–1.68)	1.64 (1.56–1.72)
Race and rheumatic disease* interaction			
AN/AI without RD	Reference	Reference	Reference
AN/AI with RD	1.11 (0.95–1.30)	2.20 (1.66–2.91)	1.25 (1.01–1.55)
White without RD	1.41 (1.33–1.51)	1.41 (1.33–1.51)	1.39 (1.30–1.48)
White with RD	0.69 (0.59–0.79)	2.11 (1.99–2.23)	1.21 (1.04–1.39)
Other without RD	2.05 (1.88–2.23)	2.04 (1.87–2.22)	2.02 (1.85–2.20)
Other with RD	1.49 (1.29–1.68)	3.87 (3.69–4.05)	1.73 (1.35–2.10)

*With or without rheumatic disease (RD) refers to the presence or absence of the specific disease in the column heading for each model (i.e. osteoarthritis, gout, or other rheumatic disease). As in previous tables, “Other Rheumatic Disease” includes people with a diagnosis of rheumatoid arthritis, lupus/MCTD, spondyloarthritis, vasculitis, Sjögren syndrome, systemic sclerosis, and/or myopathy. Some persons included also have osteoarthritis in addition to one or more of these conditions. Persons with gout are excluded from this category regardless of other conditions

All models include interaction terms between race and rheumatic disease status. For people with osteoarthritis alone compared to those with no rheumatic disease, no racial group had any statistically significant increase in odds of cardiovascular disease hospitalization; indeed, the odds of cardiovascular disease hospitalization were lower among people in the subgroups of both White and Other race in the presence of osteoarthritis vs. absence. For people with gout, the odds of cardiovascular disease hospitalization were higher for all racial groups compared to people of the same racial group without gout in the multivariable model. For people with other rheumatic diseases, having a rheumatic disease (compared to no rheumatic disease diagnosis) increased the odds of cardiovascular disease hospitalization among AN/AI people but not in people of White or Other race.

## Discussion

In this study, we found that having different rheumatic disease diagnoses stated during hospitalization had different associations with cardiovascular hospitalization. Specifically, having a diagnosis of gout was associated with an increased likelihood of cardiovascular disease hospitalization overall and in each racial group. In contrast, osteoarthritis was associated with lower likelihood of cardiovascular disease hospitalization than having no rheumatic disease diagnosis. Having another rheumatic disease (most commonly RA or SLE/MCTD) was associated with increased likelihood of cardiovascular disease in those of younger age and AN/AI race. While gout was associated with an increased likelihood of hospitalization for all cardiovascular conditions studied, the type of cardiovascular disease most strongly associated with other non-osteoarthritis rheumatic diseases was heart failure. On multivariable analysis controlling for other associated factors and interaction terms, there remains an association between gout and cardiovascular disease hospitalization in people of all races and between other non-osteoarthritis rheumatic disease diagnosis and cardiovascular disease hospitalization in people of AN/AI race but not people of White or Other race.

Most studies of the association of rheumatic disease with cardiovascular disease focus on one specific rheumatic disease. The findings of our study are consistent with previous studies showing high rates of cardiovascular disease risk in gout [18] and vasculitis [19]. Similar associations have been found in studies of RA, SLE, and other conditions, [1, 4] though our findings were more variable depending on age and race. While many studies have focused on acute MI and stroke, our finding that heart failure hospitalization is associated with a non-osteoarthritis rheumatic disease diagnosis is also consistent with studies in many rheumatic diseases, including RA and gout [2, 20]. 

Our finding that the association of rheumatic disease diagnosis and cardiovascular disease hospitalization varies by race is novel. Other studies have documented disparities in cardiovascular disease incidence or mortality in the general population, including higher incidence in Black compared to White populations in the U.S., but few have evaluated these disparities in association with rheumatic disease. One study found no difference in heart failure hospitalizations in Black compared to White patients with gout [20]. Disparities in cardiovascular disease incidence and mortality have been described in Indigenous North American populations, with a higher cardiovascular disease burden and mortality in American Indian and Alaska Native populations overall in the U.S [21, 22]. and in indigenous peoples of Canada [23], in contrast to the lower incidence and mortality in Alaska Native people in Alaska. Thus, the effect of rheumatic disease on cardiovascular disease risk may be different in other Indigenous North American populations from what we observed in this study in Alaska. It should be noted that while disparities exist by race as self-reported on hospital admission, race is not a biologic construct and it is likely that other factors such as social determinants of health are the underlying reason for such disparities.

Our finding that having a diagnosis of osteoarthritis was associated with lower likelihood of cardiovascular disease hospitalization was unexpected. While we did not expect osteoarthritis to confer a very high risk of cardiovascular disease, other studies have found a small increase in risk and a meta-analysis of observational studies confirmed a 24% increase in risk of cardiovascular disease associated with osteoarthritis [24]. In our recent study of hospitalized infections using this same data source, we found a similar negative association between osteoarthritis and hospitalized infection [15]. We hypothesize that people who are hospitalized in Alaska with a diagnosis of osteoarthritis may be healthier overall than people hospitalized for other conditions. Elective joint replacement surgery did account for at least a quarter of hospitalizations for patients with a diagnosis of osteoarthritis, and we plan to investigate the characteristics of people with joint replacement surgery in future research.

This study has some limitations. First, we used an existing dataset that included inpatient data only and identified rheumatic disease diagnoses based on inpatient coding. Because corresponding outpatient data were not available, we were not able to analyze a population-based cohort of all people with rheumatic diseases. It is possible that some patients had a rheumatic disease but did not have the diagnosis indicated in their hospital encounter. This may have led to selection bias towards more severe cases of rheumatic disease, where the diagnosis may have been more readily apparent to physicians. In contrast, as described above, people with an osteoarthritis diagnosis may be healthier than the general population if hospitalized for elective joint replacement surgery, which may lead to selection bias toward milder cases. There is also a possibility of collider bias, where having a cardiovascular disease hospitalization may make a physician more likely to also state the rheumatic disease diagnosis during hospitalization. Similar to the limitations in stated rheumatic disease diagnosis, comorbid chronic conditions may be more likely to be stated when patients have a cardiovascular disease hospitalization which could affect our analysis of the comorbidity index. However, these limitations are inherent in health services research and are similar to those of other studies. Second, the hospital discharge dataset did not allow for a multiracial category or gender other than male or female. While it is expected that a small proportion of the study population would be considered multiracial or have gender other than male or female, this limitation of the dataset may affect comparability to other contemporary studies. Third, for much of the data analysis we combined several types of rheumatic disease and all types of cardiovascular disease, and there may be heterogeneity between individual conditions. However, we were able to investigate specific rheumatic disease associations and specific types of cardiovascular disease in additional analyses. Fourth, the finding that non-osteoarthritis rheumatic disease increased the odds of cardiovascular disease hospitalization in AN/AI people may not be generalizable to other Indigenous North American populations with higher baseline prevalence of diabetes or cardiovascular disease. Fifth, we did not have individual patient-level data on several characteristics that could have influenced outcomes, such as medication prescription, chronic disease management, or social determinants of health. Importantly, while we did find differences by race, these should not be interpreted as biologic differences without assessment of social determinants of health. Finally, as an observational study of a hospital discharge data set, we are unable to attribute causation and can only report on cross-sectional associations. For example, we are not able to determine whether gout increases the risk of heart failure, or if heart failure increases the risk of gout. This limitation is inherent to many observational studies, and prospective studies of this specific question in AN/AI populations are needed. Given the small amount of literature on health disparities in hospitalizations for AN/AI people with rheumatic diseases, this study’s primary strength lies in beginning to fill a gap in the literature.

## Conclusions

This study confirmed the increased likelihood of cardiovascular disease hospitalization in people with gout and in certain subsets of people with other rheumatic disease diagnoses stated during a hospitalization. We identified differences based on the type of rheumatic disease and the type of cardiovascular disease. In the overall population, cardiovascular disease hospitalizations were less likely among AN/AI people in Alaska, but a rheumatic disease diagnosis increased the odds of cardiovascular disease hospitalization more among AN/AI people than those of White or other races. This adds significantly to the literature on the association between rheumatic disease and cardiovascular disease in Indigenous North American populations.

### Electronic supplementary material

Below is the link to the electronic supplementary material.

Supplementary Material 1

## Data Availability

The data that support the findings of this study are available from the State of Alaska, Department of Health and Social Services, but restrictions apply to the availability of these data, which were used under a restricted data use agreement for the current study and are not publicly available. Data are however available from the State of Alaska upon reasonable request and permission.

## Abbreviations

MI: Myocardial infarction
RA: Rheumatoid arthritis
SLE: Systemic lupus erythematosus
INA: Indigenous North American
AN/AI: Alaska Native/American Indian
HFDR: Health Facilities Data Reporting Program
ICD: International Classification of Diseases
MCTD: Mixed Connective Tissue Disease
CHF: Cardiomyopathy and heart failure
ASC: Coronary atherosclerosis and other heart disease
HTN: Hypertension complications
