# Supplementary material for: An assessment of cardiovascular disease hospitalizations and disparities by race in patients with rheumatic disease hospitalizations in Alaska, 2015–2018
Source: BMC Rheumatol. 2024 Feb 18;8:7. doi: 10.1186/s41927-024-00377-8 (PMC10874531; doi:10.1186/s41927-024-00377-8)
Supplement: Supplementary file 1 — Supplementary Material 1 [file 41927_2024_377_MOESM1_ESM.docx]

**Supplementary Table 1:** ICD-9 and ICD-10 codes used to define cardiovascular disease

| **Type of CVD** | **ICD-9 Codes** | **ICD-10 Codes** |
| --- | --- | --- |
| Acute myocardial infarction | 410.x | I21.x, I22.x |
| Acute cerebrovascular disease | 346.6x, 430, 431, 432.x, 433.x, 434.x, 436 | G43.601, G43.609, G43.611, G43.619, I60.x, I61.x, I62.x, I63.x, I66.x, R29.70x, R29.71x, R29.72x, R29.73x, R29.74x |
| Congestive heart failure, cardiomyopathy, and hypertensive heart disease | 398.91, 402.x, 404.01, 404.03, 404.11, 404.13, 404.91, 404.93, 428.x | I09.81, I11.0, I11.9, I13.0, I13.2, I50.x |
| Coronary atherosclerosis and other heart disease | 411.x, 412, 413.x, 414.x, V45.81, V45.82 | I20.x, I23.7, I24.x, I25.x, Z95.1, Z95.5, Z98.61 |
| Hypertension complications other than heart disease | 401, 403, 404.02, 404.1, 404.12, 404.9, 404.92, 405x, 437.2 | I12.0, I12.9, I13.10, I13.11, I15.x, I16.x, I67.4, N26.2 |
